# Rapid and Successful Implementation of a COVID-19 Convalescent Plasma Programme—The South African Experience

**DOI:** 10.3390/v13102050

**Published:** 2021-10-12

**Authors:** Tanya Nadia Glatt, Caroline Hilton, Cynthia Nyoni, Avril Swarts, Ronel Swanevelder, James Cowley, Cordelia Mmenu, Thandeka Moyo-Gwete, Penny L. Moore, Munzhedzi Kutama, Jabulisile Jaza, Itumeleng Phayane, Tinus Brits, Johan Koekemoer, Ute Jentsch, Derrick Nelson, Karin van den Berg, Marion Vermeulen

**Affiliations:** 1 Medical Division, South African National Blood Service, Roodepoort 1709, South Africa; cynthia.nyoni@sanbs.org.za (C.N.); avril.swarts@sanbs.org.za (A.S.); Ronel.Swanevelder@sanbs.org.za (R.S.); Ute.Jentsch@sanbs.org.za (U.J.); Derrick.Nelson@sanbs.org.za (D.N.); Karin.vandenBerg@sanbs.org.za (K.v.d.B.); 2 Medical Division, Western Cape Blood Service, Cape Town 7405, South Africa; Caroline@wcbs.co.za; 3 Operations Division, South African National Blood Service, Roodepoort 1709, South Africa; James.Cowley@sansb.org.za (J.C.); Cordelia.Mmenu@sanbs.org.za (C.M.); Munzhedzi.Kutama@sanbs.org.za (M.K.); Jabulisile.Jaza@sanbs.org.za (J.J.); Itumeleng.Phayane@sanbs.org.za (I.P.); Marion.Vermeulen@sanbs.org.za (M.V.); 4 National Institute for Communicable Diseases of the National Health Laboratory Services, Johannesburg 2192, South Africa; thandekam@nicd.ac.za (T.M.-G.); pennym@nicd.ac.za (P.L.M.); 5 MRC Antibody Immunity Research Unit, School of Pathology, Faculty of Health Sciences, University of the Witwatersrand, Johannesburg 2000, South Africa; 6 Information Technology Division, South African National Blood Service, Roodepoort 1709, South Africa; Tinus.Brits@sanbs.org.za (T.B.); Johan.Koekemoer@sanbs.org.za (J.K.); 7 Division of Clinical Haematology, Department of Medicine, Faculty of Health Sciences, University of Cape Town, Cape Town 7935, South Africa; 8 Division of Clinical Haematology, University of the Free State, Bloemfontein 9301, South Africa

**Keywords:** convalescent plasma, SARS-CoV-2, COVID-19

## Abstract

Background: COVID-19 convalescent plasma (CCP) has been considered internationally as a treatment option for COVID-19. CCP refers to plasma collected from donors who have recovered from and made antibodies to SARS-CoV-2. To date, convalescent plasma has not been collected in South Africa. As other investigational therapies and vaccination were not widely accessible, there was an urgent need to implement a CCP manufacture programme to service South Africans. Methods: The South African National Blood Service and the Western Cape Blood Service implemented a CCP programme that included CCP collection, processing, testing and storage. CCP units were tested for SARS-CoV-2 Spike ELISA and neutralising antibodies and routine blood transfusion parameters. CCP units from previously pregnant females were tested for anti-HLA and anti-HNA antibodies. Results: A total of 987 CCP units were collected from 243 donors, with a median of three donations per donor. Half of the CCP units had neutralising antibody titres of >1:160. One CCP unit was positive on the TPHA serology. All CCP units tested for anti-HLA antibodies were positive. Conclusion: Within three months of the first COVID-19 diagnosis in South Africa, a fully operational CCP programme was set up across South Africa. The infrastructure and skills implemented will likely benefit South Africans in this and future pandemics.

## 1. Introduction

SARS-CoV-2, the virus that causes COVID-19, poses a significant threat to global health. The lack of definitive treatment or widely accessible effective prevention has led many to consider COVID-19 convalescent plasma (CCP) as a potential therapeutic option. CCP refers to plasma collected from donors who have recovered from COVID-19 and, therefore likely to have produced neutralising antibodies against SARS-CoV-2 [1]. Convalescent plasma (CP) has been used successfully as a form of passive immunity for previous viral infections, including severe influenza, severe acute respiratory syndrome (SARS)-CoV, Middle East respiratory syndrome (MERS)-CoV and to some extent, Ebola virus disease [2,3,4]. It was hypothesised that the infusion of plasma with virus-specific antibodies might yield immediate passive immunity to the recipient and improve viral clearance [5].

Source plasma is a procedure whereby a donor’s plasma is collected through apheresis techniques, following which their cellular components are returned. Although hyper-immune plasma for Hepatitis B Virus (HBV) and source plasma for intravenous immunoglobulins (IVIG’s) from donors are routinely collected by apheresis and produced in South Africa, the country has not previously produced CP. With the outbreak of COVID-19 in South Africa from March 2020, the South African National Blood Service (SANBS) and the Western Cape Blood Service (WCBS) collaborated in setting up a national CCP programme. With a national footprint, facilities and processes for donor recruitment, plasma apheresis, infectious disease testing, component processing and inventory management already in place, blood services are advantageously placed to rapidly implement such CCP programmes. In a few short months, the SANBS and WCBS teams were successful in implementing a CCP programme.

Data is ever-evolving in the domain of COVID-19 treatments, and CCP has been gathering interest around the world as a potential therapeutic option, with international guidelines and publications on its production and use being released and updated continuously [6]. Of particular interest is the viral evolution and formation of new variants and the possible consequences they have on the efficacy of CCP from an alternate variant. Initially, a number of cohort studies [7,8,9] showed the effectiveness of CCP, requiring the need for large randomised control trials to establish both efficacy and safety. Our programme was intended for use in a phase III randomised controlled trial. Unfortunately, when the results of the large randomised control trial became available, the use of CCP in hospitalised patients with COVID-19 pneumonia showed little or no benefit [10,11,12,13]. However, there is evidence of clinical efficacy in specific population groups such as older, at-risk patients [14], early in the disease [15], and patients with immunosuppression secondary to haematology malignancies [16]. Introducing a new blood product programme at the height of a pandemic posed multiple challenges, including regulatory, logistical, ethical and scientific considerations [17], especially in resource-restricted settings [18]. This manuscript describes our efforts in addressing these challenges in South Africa.

## 2. Materials and Methods

### 2.1. Study Setting

SANBS and WCBS collect approximately 960,000 and 150,000 donations per annum, respectively, from a population of ~60 million people. SANBS covers 8 of the 9 provinces, and WCBS covers the Western Cape Province. The 8 provinces that SANBS services are structured into 7 collection and processing zones, with donation testing laboratories located in two of the zones. Both blood services are vein-to-vein organisations in that they collect, process, test, store and manage the compatibility testing and issuing processes of blood transfusion.

### 2.2. Regulatory Approval

Given the lack of empirical evidence on the efficacy of CCP at the start of the epidemic, the CCP programme in South Africa was initially limited to a clinical trial setting, which required multiple layers of regulatory approval. Two independent protocols were developed—one for CCP manufacturing and one for CCP clinical use; they were named the PROTECT-Donor (PROspective, randomised, placebo-controlled, double-blinded, phase III clinical trial of the Therapeutic use of convalEsCenT plasma in the treatment of patients with moderate to severe COVID-19) and PROTECT-Patient, respectively. The PROTECT-Donor protocol, which included donor recruitment, informed consent, collection, testing, processing and storage of CCP, required approval from the SANBS Scientific Review and Human Research Ethics Committees (2019/0519). The PROTECT-Patient protocol required additional regulatory authority approvals, including the South African Health Products Regulatory Authority (SAHPRA) for the use of an investigational product, Department of Health and site-specific approvals from clinical sites and registration on trials.gov (NCT04516811). The study was conducted according to the guidelines of the Declaration of Helsinki and approved by the Human Research Ethics Committee of the South African National Blood Service (protocol code 2019/0519 on the 21 April 2020).

During the COVID-19 pandemic in South Africa, lockdown and travel restrictions and regulations were instituted by the South African government controlling various activities based on alert levels 1–5 [19]. These restrictions included limited movement outside one’s primary residence, closure of all non-essential facilities and bans on the sale of alcohol. These restrictions significantly impacted the ability of the team to obtain these regulatory approvals and implement the programme.

### 2.3. Study Preparation and Staff Training

SANBS was collecting source plasma as a routine product prior to the start of the study, with an established infrastructure already in place. WCBS had not collected source plasma previously, so the placement of two apheresis machines in their Specialised Donation Headquarter Clinic in Cape Town and full training of three staff members was required. After obtaining the required regulatory approvals, detailed work instructions regarding all aspects of recruitment, collection, testing, processing and transport of CCP for the study were compiled by both blood services along with specific forms to facilitate the transport of the study samples and products to the correct locations. Study-specific training and competency assessments took place at the relevant sites, which was challenging at times due to the travel restrictions. Two nurses were recruited to manage the study and perform recruitment, and a medical technologist was assigned to assist in the management of samples at SANBS to accommodate the study workload.

### 2.4. Donor Recruitment

Several platforms were utilised to recruit participants, including media coverage through television and radio interviews and advertisements on the SANBS and WCBS websites. The tele-recruiters and customer service staff at both blood collection services were educated about the study and asked to advertise to existing donors. One targeted strategy was the involvement of ‘recruitment partners’ that included treating doctors and community ambulance organisations who encouraged eligible COVID-19 patients to consider registering as donors after they had recovered.

Participating donors were required to meet the routine criteria for source plasma collection, be between the ages of 18 to 65 years and have a confirmed positive COVID-19 PCR laboratory test. Initially, donors were required to be symptom-free for at least 28 days, although this was later reduced to 14 days to align with updated international guidelines [20]. Due to their higher rates of human leucocyte antigen (HLA)/human nuclear antigen (HNA) antibodies, previously pregnant females were initially excluded from recruitment as such antibodies are associated with a higher risk of antibody-associated transfusion-related acute lung injury (TRALI). However, a protocol amendment was approved to include previously pregnant females conditioned upon a negative HLA/HNA antibody test.

Donor registration was done through an established link on the SANBS website, which guided potential donors through a pre-screening questionnaire where they could record their contact details. The research nurses contacted the registered donors to perform a telephonic interview. Once preliminary eligibility had been confirmed, information regarding the study was provided, and telephonic consent was obtained. An appointment for a pre-screening visit was arranged with the participating donor centre most conveniently situated to the donor.

### 2.5. Donor Screening and Plasma Collection

The purpose of the initial pre-screening visit was to ensure that the donor met all physical and laboratory criteria for study participation. This involved completion of the routine blood donation questionnaire, testing of their haemoglobin levels by a quantitative point-of-care device and examination of their veins for suitability to donate plasma. Provided these criteria were met, enrolment was completed by the donor signing the informed consent document and having pre-screening blood specimens taken (routine viral and immunohaematology blood donation testing, full blood count and SARS-CoV-2 antibody titre testing). A provisional appointment was made for the donor’s first plasma donation. It was explained that the donor needed to have a sufficiently high SARS-CoV-2 binding antibody level (defined as a SARS-CoV-2 spike enzyme-linked immunosorbent assay (ELISA) antibody optical density (OD_450nm_) value > 0.4) to participate in the study and that this result would be communicated telephonically. If so, they would return to the clinic for their first CCP donation, provided routine donation questionnaire answers, and haemoglobin screening was passed on the day.

CCP donations took place at donor sites across all nine provinces and followed the same collection protocol as for source plasma. Donors were encouraged to donate plasma every two weeks, and follow-up donation dates were ideally agreed upon before the donor left the clinic on the day of donation. Routine source plasma donor screening was performed, and samples for SARS-CoV-2 binding antibody titre levels were taken at each visit. In the event that a donor’s antibody titre fell below the required value of SARS-CoV-2 ELISA spike OD_450nm_ < 0.4, they would be offered repeat testing a month later. If this result was persistently low, the donor was thanked for their participation in the study and motivated to become a regular apheresis or whole blood donor. As for other blood donors in South Africa, CCP donors were not remunerated in this study. Communication between CCP donors and the study staff was made by email, telephone and via social media applications such as WhatsApp^®^.

During all contact visits, infection prevention control (IPC) measures were followed, such as hand hygiene practices, environmental infection control and wearing personal protective equipment (PPE), including masks. All donor centres and staff adhered to the South African guidelines for quarantine and isolation in relation to COVID-19 exposure and infection [21] at all times.

### 2.6. Donor Testing

Routine blood donation testing, including transfusion transmissible infectious disease testing (TTID), was performed in-house in line with the standard blood screening algorithm using the Beckman Coulter PK7300 instrument (Brea, CA, USA) for blood grouping, ABO titres and syphilis (TPHA) testing; Sitetech Erythra instrument (Grifols, Spain) for irregular antibody screening; Abbott Alinity S instrument (Delkenhein, Germany) for HIV, Hepatitis B virus(HBV) and Hepatitis C virus (HCV serology testing; and Grifols Panther instrument (Barcelona, Spain) for individual-donation nucleic acid testing (ID-NAT) for HIV, HBV and HCV.

The National Institute of Communicable Disease (NICD) performed SARS-CoV-2 Spike ELISA antibody titre testing using an in-house assay based on the assay developed by Mount Sinai [22]. A spike OD_450nm_ of ≥0.4 was considered positive, and all donors with values higher than this were recruited for the study. Testing for neutralising antibodies (nAb) was performed by the NICD on stored samples, using a previously validated published method [23]. SARS-CoV-2 Wuhan-1/D614 spike proteins were expressed in Human Embryonic Kidney (HEK) 293F suspension cells by transfecting the cells with the spike plasmid. After incubating for six days at 37 °C, 70% humidity and 10% CO_2_, proteins were first purified using a nickel resin followed by size-exclusion chromatography. Relevant fractions were collected and frozen at −80 °C until use. Two μg/mL of spike protein were used to coat the 96-well high-binding plates and incubated overnight at 4 °C. The plates were incubated in a blocking buffer consisting of 5% skimmed milk powder, 0.05% Tween 20, 1× phosphate-buffered saline. Plasma samples were diluted to a 1:100 starting dilution in a blocking buffer and added to the plates. Secondary antibody was diluted to 1:3000 in blocking buffer and added to the plates followed by TMB substrate (Thermofisher Scientific, Waltham, MA, USA). Upon stopping the reaction with 1 M H_2_SO_4_, absorbance was measured at a 450 nm wavelength. In all instances, mAb CR3022 was used as a positive control, and palivizumab was used as a negative control. In line with published literature [20] and guidance from researchers in the clinical arm of the CCP trial, CCP was only collected from donors who had nAb titres greater than 1:160 to ensure that the therapeutic dose would be adequate. While nAb testing is believed to be superior, it is a time-consuming and labour-intensive process that requires a Biosafety Level 2 laboratory. The preliminary results of anti-spike binding antibodies and nAb showed a good correlation as in several other studies [24,25]. To ensure efficient use of available resources, a decision was taken to prioritise samples with spike OD_450nm_ values > 2 for nAb testing.

Previously pregnant females were screened for HLA Class I and II and HNA antibodies utilising One Lambda’s LabScreen Single Antigen (SA) I and II and LabScreen Multi-kits (Thermo Fisher Scientific, Waltham, MA, USA). These tests were performed on the Luminex 200 and FM3D instruments (Thermo Fisher Scientific, Waltham, MA, USA) as per the manufacturer’s instructions. A cut-off mean fluorescence intensity (MFI) value of 2000 was used for SA I and II HLA antibodies. Donors with a mean MFI > 2000 were considered high risk for TRALI [26]. The assay also provided a panel reactive antibody (PRA) result for SA I and II results. This is a common method to determine the level of sensitisation, expressed as a percentage (%). A high PRA% means that the individual is primed to react immunologically against a large proportion of the populations’ HLA antigens. The MFI and PRA% were analysed together to assess risk for TRALI.

### 2.7. Processing and Inventory Management

CCP donations collected by SANBS were transported to the zone processing site where they were blast frozen to −60 °C over 45–60 min, then sent to a centralised site for pathogen reduction treatment (PRT). Units collected by the WCBS were processed, pathogens were reduced on-site at the headquarters facility, and only CCP units that met the antibody criteria underwent PRT. At SANBS, PRT was performed using the Intercept^®^ PRT system (Cerus Corporation, Concord, CA, USA); WCBS used the Mirasol^®^ system (Terumo BCT, Lakewood, CO, USA) and performed the PRT.

Early in the study, a number of damaged CCP units were identified at SANBS during the initial thawing process. The study team worked quickly to investigate and implement changes to minimise CCP unit loss. These investigations included an interrogation of the bag specifications and suitability, removal of excess air at the time of sterility sampling, reducing freezer temperatures and run times to −30 °C and 45 min, respectively, placing bubble wrap between the plasma bag and dry ice in the transport process and seeking advice from the PRT manufacturer. Staff were instructed to take additional care when handling the units, to physically inspect all units, place them directly in bubble wrap bags and limit packaging to a maximum of ten units per crate. A different hamper was introduced for transporting units to the central processing centre to minimise movement during transport. A warm air plasma thawer replaced the water bath, which limited both breakages and potential bacterial contamination.

The pathogen-reduced CCP units were distributed as required from the processing sites at SANBS and WCBS to the different blood banks serving the hospitals participating in the clinical trial. CCP products were managed as per routine inventory procedures in the blood management system, identified by specific product codes. Routine blood ordering request forms were used by the clinical trial staff to order the products, and standard procedures for the issuing of blood products were followed. ABO blood group-specific products were selected for study patients and were thawed in the blood bank prior to issue. As the clinical trial was double-blind and placebo-controlled, the blood bank staff received instruction from the Randomisation Officer to prepare either the CCP or the saline placebo, which were wrapped to ensure the products were near indistinguishable, to issue to the study nurse.

### 2.8. Information Technology and Analytical Support

An application developed on the K2 cloud-based software (Nintex Group PTY LTD., Bellevue, WA, USA) was used to register and manage potential CCP donors, irrespective of the platform where they were recruited. All donor information, including contact details and information on each donation, were captured on this system. Once a donor arrived for a pre-donation screening, the donor was registered on the blood establishment computer system (BECS), and all subsequent donations, testing and management of processed blood products were captured as per routine core processes. The SANBS and WCBS BECS were modified to include CCP donations as well as the various products made through the processing of the donation. In addition, the BECS systems were modified to include the anti-SARS-CoV-2 test results. These data were included in the routine extraction, transformation and loading processes feeding the SANBS data warehouse. Operational reports combining data from WCBS and SANBS were developed by the Business Intelligence department for the day-to-day management of CCP donors, donations and products.

## 3. Results

### 3.1. Regulatory Requirements

The first case of COVID-19 was reported in South Africa on the 5 March 2020. The SANBS Scientific Research Committee and Human Research Ethics Committee approval for the CCP manufacture programme was granted in April 2020, and collections started on the 1 May 2020. The clinical CCP programme began later on the 30 September when SAPHRA approval was obtained. In the period between CCP manufacture approval and CCP clinical-use approval, CCP was collected, tested, manufactured, pathogen-reduced and stored, but not released to patients. The CCP manufacture program continued until March 2021.

### 3.2. Donor Recruitment and Plasma Collection

#### 3.2.1. Donor Enrolment and Recruitment

**Figure 1:**
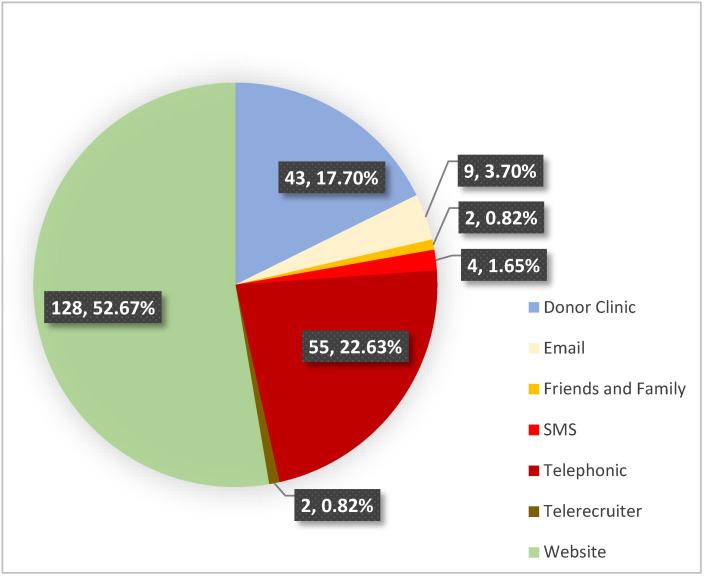
Recruitment platforms used for donor recruitment (*n*; %).

A total of 660 people expressed interest in donating CCP; of these, 156 (24%) people could not be contacted due to an incomplete registration, 157 (24%) people were excluded during the telephonic screening process and assessment appointments were scheduled for 347 (53%) people, two of whom did not attend. After routine blood donor screening, physical assessment and blood sampling, a further 104 people were excluded, mainly due to not returning for the CCP donation. A total of 243 donors were accepted for the study (Table 1). Of these, the majority (52.7%) registered on the website, followed by 22.6% making direct telephonic contact via the advertised numbers and 17.7% expressing interest at a blood donation clinic (Figure 1).

#### 3.2.2. Donor and Donation Data

The age, gender, ethnicity and blood groups of enrolled donors are shown in Table 2. Most donors were aged 20–29 years (*n* = 75; 30.9%), followed by those aged 30–39 years (*n* = 51; 21.0%) and 40–49 years (*n* = 50; 20.6%). Donors were predominantly male (*n* = 151; 62.1%), White (*n* = 178; 73.3%) and belonged to blood groups O (*n* = 102; 42.0%) and A (n = 98; 40.3%).

A total of 987 CCP units were collected from all nine provinces (Table 3). Each CCP unit comprised of approximately 650 mL and was aliquoted, when possible, into two CCP ”doses”, sufficient for approximately 1974 patients. The highest proportion of donors were recruited from the Egoli zone (35.0%), followed by the Western Cape (16.5%), with the least number of donors originating from the Free State/North Cape zone (1.2%).

The numbers of CCP donations per donor are shown in Table 4. The median number of donations made by a study participant was three, with just over a quarter of donors making only a single donation. The most donations by a study participant was 17.

#### 3.2.3. Test Results

All donations tested negative for HIV, HBV and HCV by both serological and NAT testing. One donor was excluded from the study based on a positive TPHA result.

**Figure 2:**
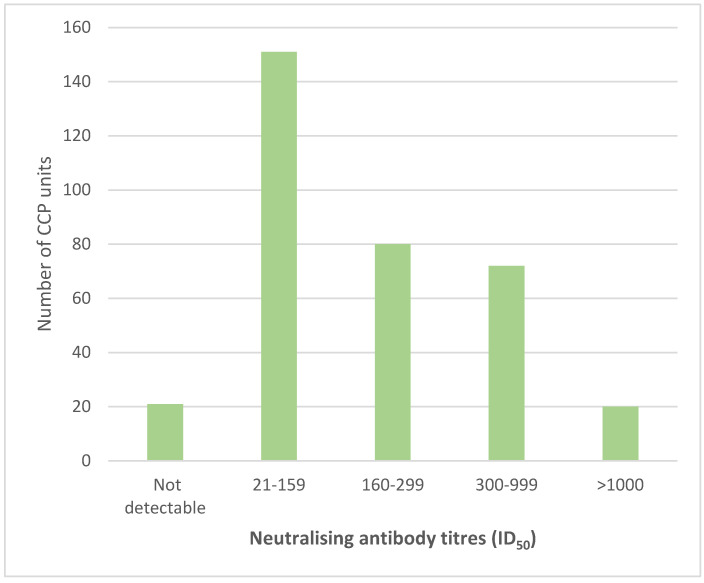
Neutralising antibody titres (ID_50_) of CCP units tested.

**Figure 3:**
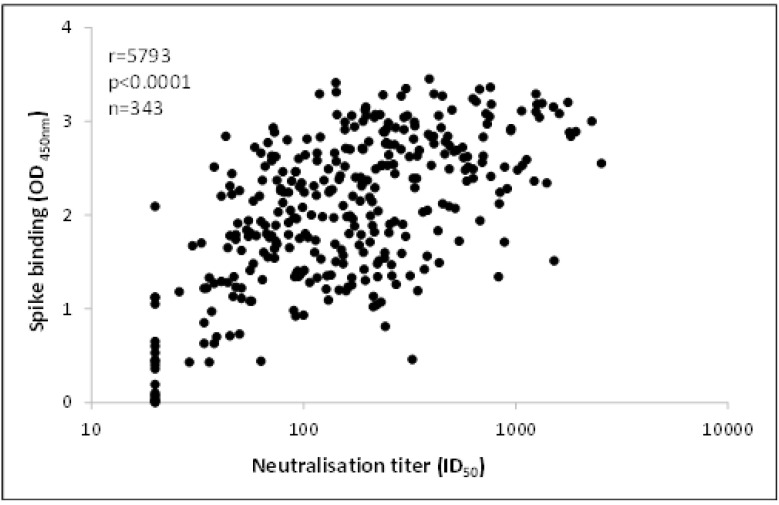
Comparison of neutralizing antibody titers and spike binding antibody responses (Optical Density at 450 nm) of CCP units.

Of the 987 CCP donations collected, 940 were tested for binding antibodies by the spike ELISA of which 54 (6%) were classified as sero-silent. In total, 886 CCP units had an OD_450nm_ ≥ 0.4 with a median (IQR) of 1.9 (1.3–2.6). The CCP units (344) were tested for nAb titres (Figure 2), of which 50% (*n* = 172) were below the cut-off of 1:160. There were 80 units (23%) with nAb titres between 160–299, 72 units (21%) with titres between 300–999 and 20 units (6%) with titres ≥ 1000. There was a good correlation between nAB titres and spike OD (Figure 3).

Anti-HNA antibodies were detected in three (8.8%) serum samples from previously pregnant donors. Anti-HLA antibodies were detected in all 34 serum samples from previously pregnant donors: 25 samples were positive for both HLA Class I and II antibodies, and the remaining nine samples were either positive for HLA Class I or Class II antibodies. The median (range) PRA% and MFI for SA I was 22% (5–52) and 2597 (993-25017), respectively. For SA II, the PRA% was 22% (1–47), and MFI was 3139 (1008-18877). When results of anti-HNA antibody, anti-HLA antibody MFI and PRA% were analysed together, all 34 samples tested were considered as high risk for causing TRALI, and these donors were excluded from donation.

## 4. Discussion

Prior to this study, CCP had not been produced in South Africa. As a novel product intended for clinical use in an acute pandemic setting, multiple, complex regulatory approvals were required, with delays and hurdles threatening the programme. To compensate for this, the programme was separated into two arms: the CCP manufacture arm (PROTECT-Donor) and the CCP clinical-use arm (PROTECT-Patient), each of which had separate protocols and approvals. Constructing the programme in this manner was time-efficient, as CCP could be manufactured while lengthy and complex regulatory approvals for clinical research use were not yet in place and time-consuming processes to find eligible hospitals with qualified doctors to take part in the clinical trial were being finalised. Within three months of the first confirmed case of COVID-19 in the country, the blood services collected the first CCP donation. Once the clinical trial was set up, the CCP product was readily available, and patient recruitment, at the height of the second wave in South Africa, was not limited by product availability.

The well-established source plasma programme at SANBS allowed for rapid implementation of a national CCP programme. Various new challenges were encountered during different stages of implementation, including logistical (ensuring staff and donor access to donor centres during the national lockdown and travel restrictions and minimising the cannibalising of whole blood donations); PPE and IPC requirements (adequate social distancing and associated decrease in the number of persons at a donation site, resulting in staggering staff shifts and reduced output); human resources (staff concerns regarding recently infected donors and reduced staff pool due to isolation and quarantine); and scientific (restricted access to and turnaround time of SARS-CoV-2 antibody testing and the rapidly evolving international recommendations of CCP titre acceptability criteria). Pathogen reduction technology had not been performed in South Africa prior to the study, and therefore procurement sourcing, installation qualification, validations, standard operating procedures and training had to be rapidly implemented.

Donor recruitment is central to a successful CCP programme. More than half of the donors contacted our research team via the website. The website was easy to navigate and provided detailed information, including the inclusion and exclusion criteria for donating, with the advantage of excluding ineligible donors upfront and negating the need for one-on-one telephonic screening.

The demographical distribution of CCP donors was similar to routine blood donors in some respects, including age and blood group. White donors were overrepresented compared to the active whole blood donor base. However, females were underrepresented; initially, all of the previously pregnant females were excluded, and the youngest age group was not well represented. This may be due to younger adults (under the age of 20) experienced milder symptoms (or asymptomatic disease) and therefore did not present for SARS-CoV-2 testing. In addition, the closure of high schools and universities during the COVID-19 lockdown periods, which are the traditional recruitment centres for young donors, may have contributed to this underrepresentation.

The HLA and HNA antibody test results were informative as they showed that, as expected, previously pregnant females had a high rate of antibody positivity. The presence and strength of these antibodies are postulated to be related to the number of previous pregnancies and time since the most recent pregnancy [27,28]. In view of the high cost and time constraint of HLA and HNA antibody testing, it may be prudent to exclude all previously pregnant females regardless of parity. This is specifically applicable in lower- and middle-income countries with limited resources and availability of specialised tests.

Collaboration and partnership with key stakeholders was a cornerstone of this programme’s success. Close collaboration between SANBS and WCBS in all aspects of this study allowed for the inclusion of donors and recipients across South Africa to donate and receive CCP in a near-seamless fashion, ensuring standardised approaches to both donor and patient selection, monitoring and reporting. Partnering with the NICD enabled access to specialised research SARS-CoV-2 antibody testing, and successful grant applications allowed for prompt funding of additional staff, equipment and testing requirements.

This study had some limitations. Data were not collected on which recruitment type was the most successful (such as radio interviews, posters, recruitment by healthcare workers), which would have been helpful for future projects. Although the website was a successful form of donor recruitment, if CCP demand had been significantly increased, the donor pool would have been insufficient to meet demands, and alternate methods of recruitment would have been required.

Of major importance, our CCP programme (PROTECT-Donor) occurred during the first wave of COVID-19 in South Africa (in which the dominant circulating viral strain was wild-type); however, the clinical arm (PROTECT-Patient) occurred during the second wave of COVID-19 in South Africa (in which the dominant viral strain was the Beta variant). The study was ended prematurely when it was discovered by colleagues at the NICD that CCP collected from donors infected with the “wild-” type virus had poor neutralising capacity against the widely circulating Beta variant [29]. SANBS and WCBS operate independently of the hospitals, national laboratory services and research-based COVID-19 surveillance programmes and therefore, definitive results of which COVID-19 variant our CCP donors were infected with was not available to us, thus hampering the success of this study.

More than half of the donors made only three or fewer donations. Although this may appear to be disappointing, neutralising antibody titres wane over time, and a robust CCP programme relies on regular donations from donors with high titre antibody levels. Testing of antibody titres at each donation allowed for donors with high titres to be actively recruited, while those with low titres were encouraged to donate whole blood or source plasma instead. This had dual benefits as the number of clinically acceptable, high titre CCP donations increased, and the routine donor pool, which was extremely constrained due to lockdown effects, also increased.

## 5. Conclusions

Despite an intense international effort, there is still a lack of both definitive treatment options and broad access to preventative options for COVID-19. The therapeutic role of CCP is yet to be fully determined, with studies showing futility [10,11,13,30] and benefit [14,16,31] in different clinical settings. The CCP dose, timing, titre, donor and patient population are still of debate, and the emergence of variants of concern complicates the testing and selection of CCP. However, what is proven is the ability of blood services to rapidly implement a large-scale nationwide CCP collection, testing, processing and release programme. Blood services are uniquely positioned to implement CCP programmes by leveraging already established systems. The skills acquired and systems implemented to achieve this programme are likely to benefit SANBS and WCBS in the current and future pandemics. In addition, this programme has created a lasting legacy of new working partnerships, which have opened new avenues of collaboration for future projects.

## Figures and Tables

**Table 1:** Donor registrations.

	Number of Donors	%
Registrations on SANBS website	660	100
Incomplete registration	156	23.6
Failed telephonic screening interview	157	23.8
Failed pre-screening assessment at donation site	104	15.8
Enrolled	243	36.8

**Table 2:** Age group, gender, blood group and ethnicity classification of enrolled donors.

		Number of Donors	%	Number of Donations	%
Total		243	100	987	100
Age Group	18–19	7	2.9	14	1.4
(years)	20–29	75	30.9	267	27.1
	30–39	51	21.0	166	16.8
	40–49	50	20.6	222	22.5
	50–59	45	18.5	244	24.7
	60–69	15	6.2	74	7.6
Gender	Female	92	37.9	349	35.4
	Male	151	62.1	638	64.6
Blood Group	Group A	98	40.3	396	40.1
	Group AB	11	4.5	47	4.8
	Group B	32	13.2	148	15.0
	Group O	102	42.0	396	40.1
Ethnicity	Asian/Indian	20	8.2	92	9.3
	Black African	22	9.1	80	8.1
	Coloured	19	7.8	87	8.8
	Unknown	4	1.7	19	1.9
	White	178	73.3	709	71.8

**Table 3:** Location of donors and total collections.

		Number of Donors	%	Number of Donations	%
Zone	Eastern Cape	27	11.1	124	12.6
	Egoli	85	35.0	370	37.5
	Free State/North Cape	3	1.2	13	1.3
	KwaZulu Natal	16	6.6	91	9.2
	Mpumalanga	10	4.1	40	4.1
	Northern	37	15.2	158	16.0
	Vaal	25	10.3	89	9.0
	Western Cape	40	16.5	102	10.3
	Totals	243	100	987	100

**Table 4:** Number of donations per donor.

Number of Donations	Donors	% of Donors
1	65	26.8
2	34	14.0
3	37	15.2
4	25	10.3
5	20	8.2
6	20	8.2
7	6	2.5
8	7	2.9
9	6	2.5
10	6	2.5
11	7	2.9
12	5	2.1
13	1	0.4
14	1	0.4
15	2	0.8
17	1	0.4

## Data Availability

The data presented in this study are available on request from the corresponding author. The data are not publicly available due to SANBS data privacy policy.
